# Crystal structures of the UDP-diacylglucosamine pyrophosphohydrase LpxH from *Pseudomonas aeruginosa*

**DOI:** 10.1038/srep32822

**Published:** 2016-09-09

**Authors:** Chiaki Okada, Hiroko Wakabayashi, Momoko Kobayashi, Akira Shinoda, Isao Tanaka, Min Yao

**Affiliations:** 1 Faculty of Advanced Life Science, Hokkaido University, Sapporo 060-0810, Japan; 2 Graduate School of Life Science, Hokkaido University, Sapporo 060-0810, Japan; 3 Department of Pharmacology, Basic Medical College of Zhengzhou University, Zhengzhou, China

## Abstract

Lipid A (also known as endotoxin) is the hydrophobic portion of lipopolysaccharides. It is an essential membrane component required for the viability of gram-negative bacteria. The enzymes involved in its biosynthesis are attractive targets for the development of novel antibiotics. LpxH catalyzes the fourth step of the lipid A biosynthesis pathway and cleaves the pyrophosphate bond of UDP-2,3-diacylglucosamine to yield 2,3-diacylglucosamine 1-phosphate (lipid X) and UMP. Here we present the structures of LpxH from *Pseudomonas aeruginosa (Pa*LpxH). *Pa*LpxH consists of two domains: a catalytic domain that is homologous to the metallophosphoesterases and a helical insertion domain. Lipid X was captured in the crevice between these two domains, with its phosphate group facing the dinuclear metal (Mn^2+^) center and two acyl chains buried in the hydrophobic cavity. The structures reveal that a large conformational change occurs at the lipid X binding site surface upon the binding/release of the product molecule. Based on these observations, we propose a novel model for lipid X embedding, which involves the scissor-like movement of helix α6, resulting in the release of lipid X into the lipid bilayer.

Lipopolysaccharide (LPS) is the main component of the outer membrane of gram-negative bacteria. Composed of a polysaccharide chain and lipid moiety, LPS forms a permeability barrier to protect gram-negative bacteria from environmental stresses, such as detergents and antibiotics. The hydrophobic portion of LPS (lipid A) anchors LPS to the outer membrane^1^. Lipid A (also known as endotoxin) is the active component of LPS and is responsible for many of the pathological disorders caused by infection with gram-negative bacteria. It strongly stimulates the innate immune system of animals and induces an inflammatory reaction in the host cells by activating the TLR4/MD2 receptor of the mammalian innate immune system^2^. Lipid A is very important for the viability and pathogenicity of gram-negative bacteria; therefore, enzymes involved in lipid A biosynthesis are attractive targets for the development of novel antibiotics.

In *Escherichia coli* lipid A is synthesized from UDP-N-acetylglucosamine (UDP-GlcNAc) in nine steps, each of which are catalyzed by different enzymes (Fig. 1)^3^. Most of these constitutive enzymes are well conserved in gram-negative bacteria. In the fourth step in this pathway, UDP-2,3-diacylglucosamine is hydrolyzed to 2,3-diacylglucosamine-1-phosphate (also called lipid X) and UMP. This step is catalyzed by the specific pyrophosphatase LpxH in 70% of gram-negative bacteria, including *E. coli* and *Pseudomonas aeruginosa*, and by LpxI in the remaining 30%^3^,^4^,^5^. Although these two enzymes provide the same product from the same substrate, they have no sequence similarity and are not found in the same organisms. Furthermore, they have different metal ion requirements and attack different phosphorus atoms during hydrolysis; LpxH attacks the α-phosphate of UDP-2,3-diacylglucosamine, and LpxI attacks the β-phosphate^4^. In spite of these differences, LpxI can compensate for LpxH loss in *E. coli*; thus, the two enzymes are considered to have the same role in the context of lipid A biosynthesis^4^.

The tertiary structure of LpxI is known^5^, but the structure of the major isozyme LpxH has not been studied to date. LpxH shares sequence similarity with metallophosphoesterases and requires Mn^2+^ ions for phosphatase activity^6^. To provide insight into the reaction mechanism of this important peripheral membrane protein and lipid X biosynthesis, we have determined the structure of LpxH from *P. aeruginosa (Pa*LpxH). In the present study, we analyzed five different crystal structures, including the product complex (*Pa*LpxH–lipid X) with and without Mn^2+^ ions. Although wild-type *Pa*LpxH captured lipid X from *E. coli* cells, the H10N mutant, which has lost Mn1-binding ability, captured neither lipid X nor the substrate. Moreover, LpxH undergoes a large conformational change upon release of lipid X, which suggests that the membrane embedding of the product molecule is different from the one proposed for LpxI. More recently, as we prepared this manuscript, a third constitutive UPD-2,3-diacylglucosamine hydrolase, LpxG, was found in the genomic library of *Chlamydia trachomatis*^7^. The relationship of this constituent member with LpxH is also discussed.

## Results

### Structure analysis of *Pa*LpxH

**Figure 2:**
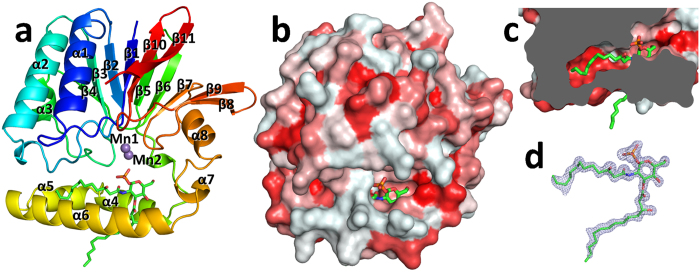
Structure of *Pa*LpxH. (**a**) Ribbon diagram of the *Pseudomonas aeruginosa* LpxH (*Pa*LpxH) shown in rainbow color (*P*2_1_ crystal with Mn^2+^). Lipid X is depicted by green sticks and the Mn^2+^ ions are violet spheres. *Pa*LpxH consists of a catalytic domain of approximately 180 residues (Met1–Leu118 and Val174–Leu240) and a helical insertion domain (HI domain) (α4–α7). (**b**) Surface representation of the *Pa*LpxH. The structure is colored in red with different density based on the hydrophobicity scale^30^. (**c**) Cross-sectional view of the *Pa*LpxH focusing to hydrophobic cavity between the catalytic and HI domains. The 2-acyl chain of the lipid X is deeply buried in the cavity. d. Fo–Fc omit maps superposed with bound lipid X (cut off 3.5 σ).

*Pa*LpxH was crystallized in the presence of Mn^2+^ ions, which are required for enzymatic activity^6^,^8^. The structure was determined with the multi-wavelength anomalous diffraction method using Mn^2+^ ions. Two *Pa*LpxH molecules were present in the asymmetric unit of the *P*2_1_ crystal, and all 239 amino acid residues were unambiguously determined for both molecules (Fig. 2a). Furthermore, the high-resolution electron density (1.65 Å) clearly identified a stoichiometrically bound product molecule (lipid X, 2,3-diacylglucosamine-1-phosphate) and two Mn^2+^ ions in each *Pa*LpxH molecule (Fig. 2). The lipid X molecule was likely captured in *E. coli* cells because no substrate molecule (UDP-2,3-diacylglucosamine) or product molecule (lipid X) was added during purification or crystallization. Proteins without Mn^2+^ ions were also successfully crystallized in two different space groups. A *C*2 form was obtained with no Mn^2+^ ions added during crystallization, and a *P*2_1_ form was obtained by adding EDTA prior to crystallization. These crystals also contained two *Pa*LpxH molecules in the asymmetric unit. The two molecules in the asymmetric unit had similar hydrophobic contacts with each other in all these crystals (see below). No major changes were observed in the protein structures regardless of the presence of Mn^2+^ ions (Fig. S1), suggesting that Mn^2+^ ions do not have a major effect on the protein structure.

**Figure 3:**
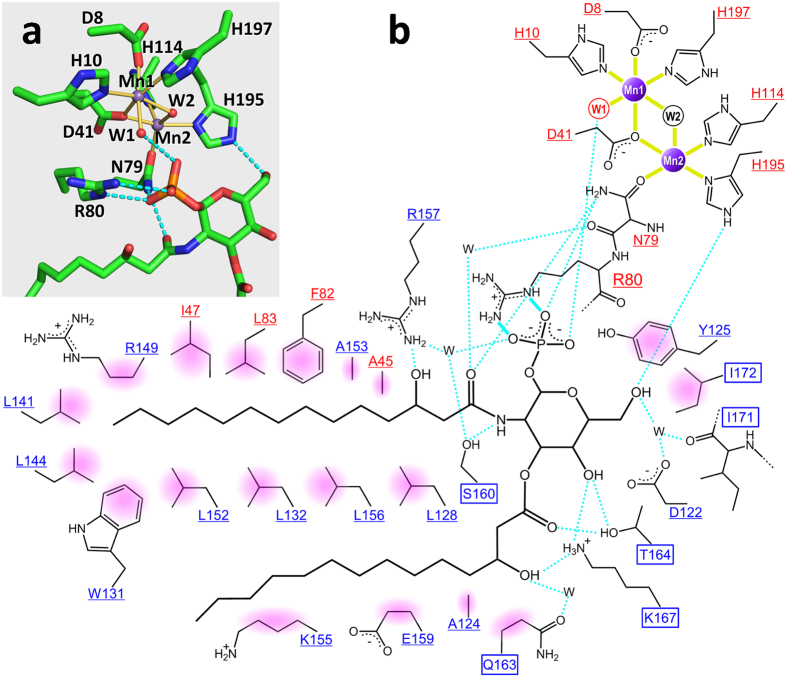
Detailed representation of Mn^2+^ and lipid X recognition by *Pa*LpxH. (**a**) Residues involved in Mn^2+^ coordination and the binding of the glucosamine-1-phosphate moiety of lipid X are shown. Mn^2+^ coordination is depicted with yellow bonds and polar interactions are depicted with light blue dotted lines. Mn1 is in octahedral coordination with six ligands, whereas Mn2 has five ligands with one open site facing the phosphate group of lipid X. Water molecules (W1 and W2) are shown as red spheres. (**b**) Schematic overview of Mn^2+^ and lipid X binding. Residues in the catalytic domain are shown in red and those in the HI domain are shown in blue. Residues whose structures change upon lipid X binding are highlighted in squares, whereas residues whose structures are unchanged are underlined. Coordination bonds are indicated with solid yellow lines, bidentate salt bridges are indicated with light blue solid lines, and hydrogen bonds are indicated with light blue dotted lines. Apolar interactions are shown with pink shading.

*Pa*LpxH consists of two domains: a catalytic domain homologous to metallophosphoesterases (MPEs) and a helical insertion domain (HI domain) inserted in the middle of the catalytic domain. The catalytic domain of approximately 180 residues (Met1–Leu118 and Val174–Leu240) is composed of two facing β sheets (a six-stranded β sheet that includes β1–β4 and β10–β11 and a five-stranded β sheet that includes β5–β9) and four peripheral α helices (α1–α3 and α8). The HI domain is composed of four α helices (α4–α7), and no similar structure was found in the PDB. At the boundary between the catalytic and HI domains, invariant residues and two Mn^2+^ ions form a dinuclear metal center (Fig. 3a).

### Recognition of lipid X by LpxH

Lipid X is positioned in a crevice between the catalytic and HI domains, with the phosphate group facing the dinuclear metal center and two acyl chains buried in the hydrophobic cavity (Fig. 2). Twenty-one amino acid residues in the HI domain and seven amino acid residues in the catalytic domain are involved in the binding of lipid X through direct or water-mediated interactions (Fig. 3b). The glucosamine-1-phosphate moiety is specifically recognized through hydrophilic and hydrophobic interactions with a number of residues that belong to both HI and catalytic domains. Residues Asn79 and His195, which are involved in Mn^2+^ coordination, also participate in the binding of lipid X (Fig. 3).

This product binding mode is quite different from that of LpxI, an enzyme that has the same catalytic activity as LpxH. In the LpxI–lipid X complex, only acyl chains are enveloped by the hydrophobic pocket of the LpxI molecule, and the glucosamine-1-phosphate moiety is positioned far out into the solvent. The two acyl chains are not distinguished, and the molecule is bound with two alternative conformations rotated by 180° about the long axis between the two acyl chains^5^. In contrast, the head region of the lipid X molecule, which is rich in hydrophilic functional groups, remains in contact with LpxH, and the two acyl chains are distinctively recognized. The 3-acyl chain interacts with LpxH only at the proximal region of the chain, with the distal part out of the LpxH molecule, whereas the 2-acyl chain is deeply buried in the hydrophobic cavity between the catalytic and HI domains (Fig. 2). Therefore, the area of interaction of the LpxH with 2-acyl chain is much larger than that with the 3-acyl chain. Most o f the interactions observed in the enzyme–product (EP) complex are likely to be present in the specific binding that occurs when LpxH recognizes the substrate. Indeed, this structure explains previous observations that the precursor molecules of UDP-2,3-diacylglucosamine, UDP-GlcNAc or UDP-3-O-(R)-3-hydroxymyristoyl-GlcNAc cannot be the substrate of LpxH^6^.

### Structural analysis of the H10N mutant (apo form)

To investigate enzymatic reaction mechanism, a mutant was prepared in which Mn1-coordinated His10 was replaced with Asn (H10N). Its structure was determined with (Mn2 bound form) and without (Mn2 unbound form) manganese ion. Both these mutants crystallized in the space group *P*2_1_2_1_2_1_. As expected, the H10N mutant did not bind to Mn1. Previous knowledge of the reaction mechanism of MPEs suggested that the mutant deficient of Mn1 binding would have no enzymatic activity^8^,^9^,^10^. Although it was prepared using the same protocol as the wild type, this *Pa*LpxH mutant did not capture a product molecule (lipid X). The substrate molecule UDP-2,3-diacylglucosamine was also not observed in the electron density map. However, except for the loss of Mn1, no large conformational change occurred in the vicinity of the mutated Asn10 residue, and the structure of the whole catalytic domain was similar to that of the wild type. Thus, the direct effect of H10N mutation on *Pa*LpxH seems to be the loss of Mn1-binding ability. Together with the fact that Mn^2+^ ions have little influence on the structure of *Pa*LpxH, we assumed that this structure represents the conformation of the enzyme when the substrate is not bound. Hereafter, we will call this structure the apo form.

### Comparison of the apo and EP complex forms

**Figure 4:**
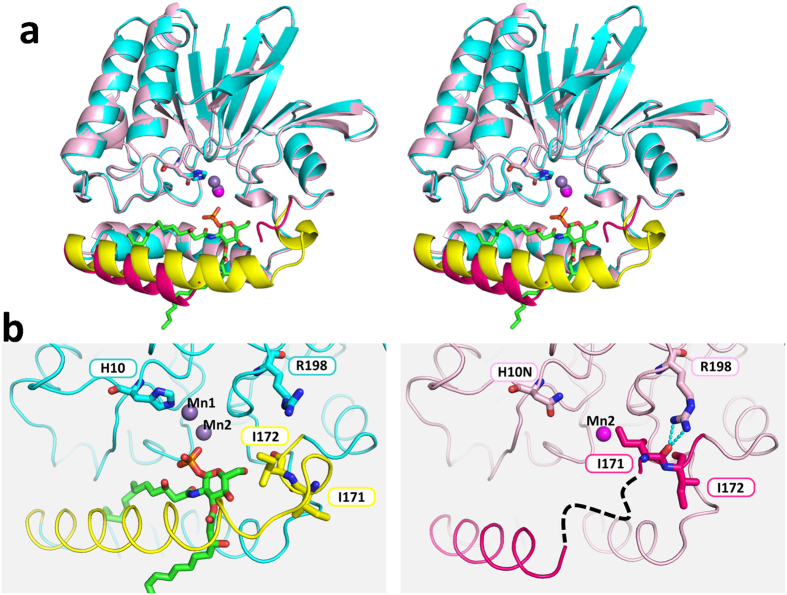
Conformational changes of *Pa*LpxH upon lipid X binding. (**a**) Stereo view of the superimposed structures of the *Pa*LpxH–Lipid X complex (cyan and yellow) and the apo form (H10N mutant, pink and red). With respect to the *Pa*LpxH–Lipid X complex, the region that changes (α6–α7, residues 146–173) upon lipid X binding is shown in yellow. Lipid X is shown in stick and Mn^2+^ ions are violet spheres. With respect to the apo form, the region that changes (residues 146–173) is shown in red. H10N is shown in stick. Mn1 is not bound, and Mn2 is shown as a pink sphere. The C-terminal half of α6 and the following loop (residues 161–169 in *P*2_1_2_1_2_1_ with Mn2, 159–165 in *P*2_1_2_1_2_1_ with no Mn^2+^)) were disordered. Helix α7 became a loop (*P*2_1_2_1_2_1_ with Mn2) or a 3_10_ helix (*P*2_1_2_1_2_1_ with no Mn^2+^). The H10N mutation does not affect the structure of the catalytic domain, whereas the structure of the α6–α7 region changes. The α6–α7 region is wide open in the apo form, whereas in the EP complex, this gate is closed, possibly by interactions with the product molecule. (**b**) Detailed views of the variable region; EP complex (left) and apo form (right). The main chain conformation at I171 and I172 changes (psi angles are changed by 180°). In the apo form, the carbonyl oxygen of I172 makes a hydrogen bond with Arg198, and I171 and I172 are positioned closer to the catalytic domain. In the EP complex, the residues in α6–α7 interact with lipid X and are fixed. As a result of the conformational change, I171 and I172 interact with lipid X, not with Arg198.

Although no major conformational differences are observed in the catalytic domain when comparing the apo and EP complexes, the structure of HI domain varies extensively in the region of α6–α7 (residues 158–172) (Fig. 4). There is no electron density for the C-terminal half of α6 and the following loop region (residues 161–169 in *P*2_1_2_1_2_1_ crystal with one Mn^2+^, 159–165 in *P*2_1_2_1_2_1_ crystal with no Mn^2+^) in the apo form, indicating they are highly mobile and disordered. Furthermore, the folding of the protein is completely changed in the subsequent region. In the apo form, α7 (residues 169–172) disappears and a loop (*P*2_1_2_1_2_1_ with one Mn^2+^) or a short 3_10_ helix at residues 168–170 (*P*2_1_2_1_2_1_ with no Mn^2+^) is created. As a result, the orientation of the side chains of residues 170–172 is completely different between the two forms. The hydrogen bonds between the side chain of Arg198 and the carbonyl oxygen of Ile171 stabilize the structure in the apo form, whereas they are not present in the EP complex due to an approximate 180° psi rotation at Ile171 and Ile172 (Fig. 4b). As shown in Fig. 3b, a significant structural change in residues 158–172 is important for the recognition of lipid X (and probably also the substrate); the binding site is wide open in the apo form, whereas in the EP complex, two alpha helices (α6 and α7) are formed to assemble a stable EP complex.

## Discussion

Members of the MPE superfamily have seven well-conserved residues coordinated to the two metal ions of the dinuclear metal center^9^,^11^,^12^. These residues (Asp8, His10, Asp41, Asn79, His114, His195, and His197) are also present in *Pa*LpxH. Six ligands [Asp8, His10, Asp41 (bridging two Mn^2+^ ions), His197, and two hydroxide ions (designated as W1 and W2)] are octahedrally coordinated with Mn1. In contrast, five ligands provide an incomplete octahedral coordination with Mn2: Asp41, Asn79, His114, His195, and W2 (bridging two Mn^2+^ ions) (Fig. 3a). In many MPEs studied to date, octahedral coordination of both metal ions of the dinuclear metal center is completed by the coordination of the phosphate group of the substrate. In terms of *Pa*LpxH, this corresponds to the coordination of the oxygen atom of the phosphate group at the vacant position of Mn2. In the ES complex, the alpha phosphate of the substrate (UDP-2,3-diacylglucosamine) is expected to occupy this position and complete the octahedral coordination of both Mn^2+^ ions.

In contrast to the catalytic domain, the structure of the HI domain is unique; Dali server^13^ detected no homologous structures in the PDB. On comparing the three-dimensional structures and topologies among the three MPEs [LpxH, Mre11 (multifunctional nuclease involved in DNA repair)^9^, and Rv0805 (cyclic AMP phosphoesterase)]^14^ (Fig. S2), these MPEs consist of a homologous catalytic domain and a specific substrate recognition domain. The HI domain is unique not only in amino acid sequence and tertiary structure but also in the relative positions of the primary and tertiary structure in the catalytic domain (Fig. S2). It is inserted into the approximate center of the catalytic domain, and the product molecule (lipid X) is trapped in the hydrophobic cavity formed at the catalytic–HI domains interface. For Mre11, peptide chains are partly inserted near the C-terminus of the catalytic domain and partly attached to the C-terminal end^9^. Rv0805 forms a functional homodimer, thereby creating an active site pocket between the two monomer units^14^. In this manner, the inserted or attached domains determine the substrate and reaction specificity of the MPEs.

**Figure 5:**
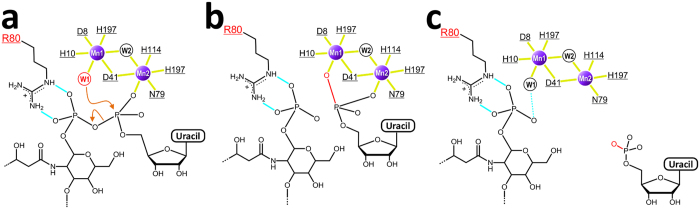
Proposed reaction mechanism. (**a**) Proposed enzyme–substrate complex. An oxygen atom of the α-phosphate of the substrate is coordinated to Mn2 (yellow line), and the β-phosphate forms a bidentate salt bridge with Arg80 (cyan lines). As a result, the phosphorus atom of the α-phosphate group is positioned close enough to be attacked by the hydroxide ion (W1, in red). (**b**) Nucleophilic attack of the hydroxide ion yields UMP and lipid X. (**c**) Observed enzyme–product complex. UMP has left and a new water molecule (W1, in black) coordinates to Mn1. A bidentate salt bridge between Arg80 and the phosphate group of lipid X is formed.

As described above, most of the interactions observed in the EP complex are assumed to be present in the ES complex as well. In the proposed hydrolysis mechanism of the Mre11 nuclease, the hydroxide ion activated by Mn1 undergoes a nucleophilic attack on the phosphorus atom of the phosphodiester linkage^9^,^15^. This hydroxide ion corresponds to the hydroxide ion W1 in the EP complex of *Pa*LpxH (Fig. 5). W1 is positioned close enough to attack the alpha phosphate of the substrate in the ES complex, as deduced from the structure of the EP complex (Fig. 5). Thus, the present structure is consistent with the previously proposed reaction mechanism^6^.

It was previously reported that six alanine mutants of *Haemophilus influenza* LpxH (*Hi*LpxH) have shown a significant decrease in hydrolysis activity^8^. Our structures show that five out of the six residues (Asp9, His11, Asp42, His115 and His196 of *Hi*LpxH; corresponding to Asp8, His10, Asp41, His114 and His195 of *Pa*LpxH) participate in Mn^2+^ coordination, and the last one (Arg81 of *Hi*LpxH; Arg80 of *Pa*LpxH) is involved in lipid X binding (Fig. 3). In many MPEs, the position occupied by Arg80 in the tertiary structure of *Pa*LpxH is occupied by the His residue involved in phosphoester bond hydrolysis. For example, the corresponding residue (His85) of Mre11 has been suggested to act as a proton donor in the enzymatic reaction^15^, and His98 of Rv0805 contributes to the stabilization of reaction intermediates during cAMP hydrolysis^14^. In other MPEs, the corresponding His residue is well conserved, and there are many reports that suggest the importance of this residue^10^,^16^,^17^. Thus, it is significant that only in LpxH, this His residue is substituted by Arg in all species of known sequence^18^ (Fig. S3). In the EP complex, the phosphate group of lipid X (corresponding to the beta phosphate moiety of UDP-2,3-diacylglucosamine) is strongly bound to Arg80 through a bidentate salt bridge. Thus, we speculate that Arg80 helps neutralize the negative charge of the ES complex (Fig. 5). LpxH exhibits a sharp decrease in its catalytic activity at low pH^6^,^8^. Using pH-specific activity rate profile of *Hi*LpxH, a p*K*_a_ of the reaction was estimated to be 6.6 ± 0.4^8^. It is in good agreement with p*K*_a_ values (~6.5) for the second ionization of pyrophosphate in UDP-GlcNAc^19^ and is consistent with the model in Fig. 5. We assume that LpxH requires both interactions of the deprotonated phosphate groups (Mn^2+^ coordination of the alpha phosphate, and salt bridge of the beta phosphate to Arg80) to form stable ES complex. The knowledge of this unique recognition mechanism of glycolipid by LpxH may facilitate the development of novel antibiotics targeting lipid A biosynthesis.

Two paralogs (designated *Pa*LpxH and *Pa*LpxH2) of *E. coli* LpxH have been shown to be present in *P. aeruginosa. Pa*LpxH2 is less similar (sharing 28% identity and 39% similarity) to *E. coli* LpxH, compared with *Pa*LpxH (sharing 46% identity and 61% similarity)^20^. *Pa*LpxH has been proven to have UDP-2,3-diacylglucosamine pyrophosphatase activity^6^, whereas no concrete evidence of the enzymatic activity of *Pa*LpxH2 has been reported. Although *Pa*LpxH2 has an insertion sequence of a size similar to that of *Pa*LpxH within its MPE domain, conserved residues are completely different between these two insertion sequences. Most of the residues that are critical for lipid X recognition or that are involved in inter-domain interactions in *Pa*LpxH are replaced by other residues in *Pa*LpxH2 (Fig. S4). This suggests that the substrate of *Pa*LpxH2 is something other than UDP-2,3-diacylglucosamine. After LpxH and LpxH2 diverted from a common ancestral protein by gene duplication, they have evolved independently to acquire different substrate specificities. The insertion sequence of *Pa*LpxH2 has a remarkably high proportion of aromatic side chains, which presumably reflects the characteristics of the substrate to be bound. Very recently, an ortholog of LpxH designated LpxG has been reported from the genomic library of *C. trachomatis*^7^. This has been proven to have UPD-2,3-diacylglucosamine hydrolase activity *in vivo* and *in vitro*. Sequence comparison clearly shows that LpxG also has an MPE domain (Fig. S4). At least six of the seven important residues coordinated to Mn^2+^ ions are conserved in LpxG. However, the residues corresponding to the helical insertion are not well conserved in LpxG. Thus, we assume that these two proteins have a distantly related common ancestor, but have independently evolved. The interfacial catalytic mechanism discussed in the next section will likely differ between these two proteins.

Two unrelated enzymes, LpxH and LpxI, are known to have the same catalytic activity of UDP-2,3-diacylglucosamine pyrophosphatases in the lipid A synthetic pathway. The present analysis shows that whereas the relative position of the catalytic and lipid-binding domains in LpxI is significantly changed when the substrate/product binds^5^, no change is observed in the relative position between the apo form and EP complex in LpxH. Rather, a significant change is observed within the HI domain (Fig. 4). Thus, although the two enzymes have the same UDP-2,3-diacylglucosamine pyrophosphatase activity and play the same role in the lipid A synthetic pathway^4^, their catalytic mechanisms are completely different.

**Figure 6:**
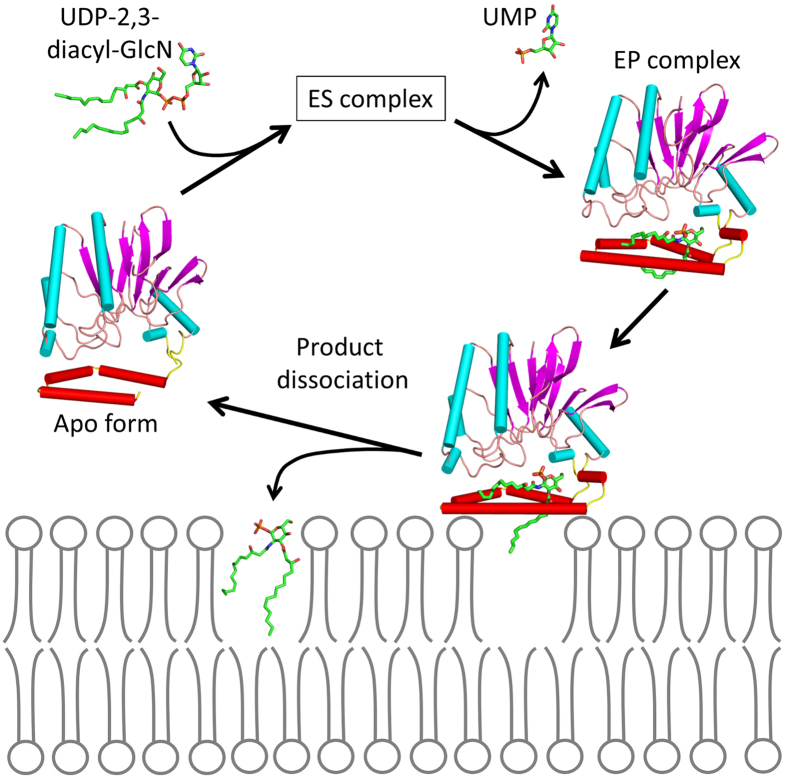
Proposed model for the interfacial catalytic mechanism. LpxH captures UDP-2,3-diacyl-GlcN and forms an ES complex in which the substrate is bound in a manner similar to that seen in the EP complex. The hydrolysis reaction occurs as in Fig. 5, whereas the hydrophobic surface of the HI domain of the EP complex is in contact with the inner membrane. The interactions between the HI domain and membrane surface induce a conformational change (or opening) in helix α6, which releases lipid X with concomitant embedding of the molecule.

An interfacial catalysis model has been proposed for LpxI in which each of the two domains forms the halves of an inter-domain active site^5^. Clearly, the structure of LpxH suggests that this scheme cannot be applied to LpxH, as no substantial change in inter-domain orientation is expected. In this regard, it is important to note that all five crystal forms solved in the present study have two LpxH molecules in the asymmetric unit and that they are packed with the same hydrophobic surface facing each other (Fig. S5). This hydrophobic surface is the outer surface of the HI domain within which the product molecule (lipid X) is bound. We suggest that this hydrophobic contact mimics the interactions between LpxH and the membrane. It has been previously reported that the addition of MnCl_2_ to the buffers used to prepare the extracts of the overproduced LpxH increases the membrane localization of LpxH hydrolase activity^6^. We assume that this increase in membrane affinity is the result of an increase in EP complex formation caused by MnCl_2_, with concomitant structural change as observed in the present study. A detailed structural comparison of the apo form with the EP complex shows that most of the important interactions between the catalytic and HI domains occur within the N-terminal half of HI domain (α4 and α5). Upon the release of lipid X, only the C-terminal half (α6 and α7) moves, whereas the N-terminal half remains unchanged (Fig. S6). Based on these observations, we propose a novel model for lipid X embedding (Fig. 6), which involves the scissor-like movement of α6 helix upon interaction with the membrane, resulting in the release of the product molecule into the lipid bilayer.

## Materials and Methods

Cloning, expression, purification and crystallization were described in supporting information.

### X-ray data collection and structure determination

All data were collected under cryogenic conditions (100 K) after crystals were flash-frozen in a gaseous nitrogen stream. Multi-wavelength anomalous diffraction (MAD) data were collected using a single *P*2_1_ crystal containing lipid X and two Mn^2+^ using a wavelength of 1.8925 Å (peak) and 1.0000 Å (remote) based on the absorption edge of the manganese atom. All data sets were indexed, integrated, scaled, and merged using the *HKL2000* program suite^21^. The structure was determined by MAD phasing using Mn^2+^ bound to LpxH as scatterers. The sites of four Mn^2+^ ions in the asymmetric unit were found using the program *SHELXD*^22^, and the initial phase was calculated and modified with the program *SHELXE*^22^. The initial model was built to 98% of *Pa*LpxH molecules using *ARP/wARP*^23^. Remaining protein residues and lipid X molecules were manually located in *Coot*^24^. Completion of the structures was straightforward using iterative cycles of manual model fitting in *Coot* and computational refinement in *REFMAC5*^25^,^26^. The structures of the other four crystals were determined using the molecular replacement method with the program *MOLREP*^27^, using the monomer structure of *P*aLpxH as a search model followed by a rigid-body refinement with the program *REFMAC5*. After completion of the refinement, the program *PROCHECK*^28^ was used to assess the quality of the final models. Images of the structures and electron density maps were generated using *PyMOL*^29^. Data collection and refinement statistics are presented in Table S1.

## Additional Information

**Accession codes:** The coordinates and structure factor amplitudes have been deposited in the Protein Data Bank (www.pdb.org). PDB ID codes: 5B49 (LpxH complexed with lipid X and Mn2+), 5B4A (LpxH complexed with lipid X (P21 form), 5B4B (LpxH complexed with lipid X (C2 form). 5B4C (LpxH mutant H10N complexed with Mn2+), and 5B4D (LpxH mutant H10N).

**How to cite this article**: Okada, C. *et al*. Crystal structures of the UDP-diacylglucosamine pyrophosphohydrase LpxH from *Pseudomonas aeruginosa. Sci. Rep.* **6**, 32822; doi: 10.1038/srep32822 (2016).

## Supplementary Material

Supplementary Information

## Figures and Tables

**Figure 1:**
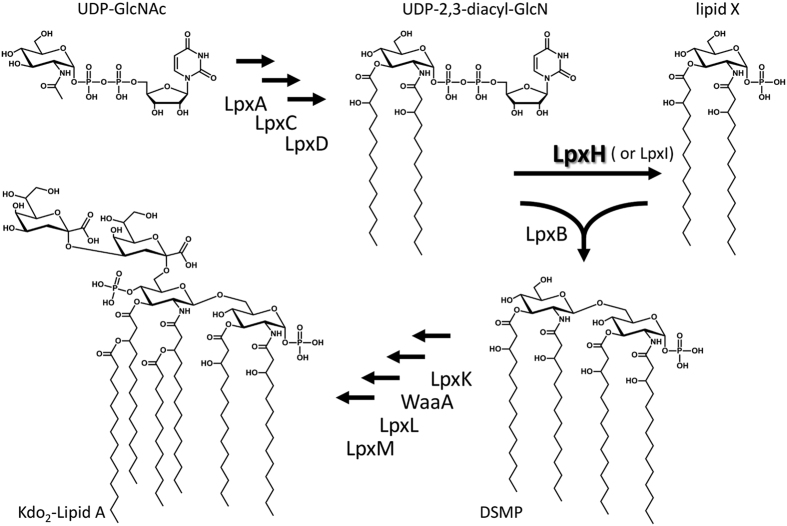
Kdo2-Lipid A biosynthetic pathway in *E. coli*^3^. Kdo_2_-Lipid A synthesis from UDP-N-acetylglucosamine (UDP-GlcNAc) is catalyzed by nine enzymes. The fourth step of the pathway involves the hydrolysis of a pyrophosphate moiety of UDP-2,3-diacylglucosamine (UDP-2,3-diacyl-GlcN) and yields UMP and 2,3-diacylglucosamine-1-phosphate (lipid X). The enzyme responsible for this hydrolysis reaction is the specific pyrophosphatase LpxH. In a few bacteria, this reaction is catalyzed by LpxI^4^. In the fifth step, LpxB condenses UDP-2,3-diacyl-GlcN with lipid X to form 2′,3′-diacylglucosamine-(β,1′-6)-2,3-diacylglucosamine 1-phosphate (DSMP).
