# Supplementary material for: Crystal structures of the UDP-diacylglucosamine pyrophosphohydrase LpxH from Pseudomonas aeruginosa
Source: Sci Rep. 2016 Sep 9;6:32822. doi: 10.1038/srep32822 (PMC5016852; doi:10.1038/srep32822)
Supplement: Supplementary Information [file srep32822-s1.pdf]

Supporting information

Crystal structures of the UDP-diacylglucosamine pyrophosphohydrase LpxH from *Pseudomonas aeruginosa*

Chiaki Okada[1], Hiroko Wakabayashi[1], Momoko Kobayash[2], Akira Shinoda[2], Isao Tanaka[1], & Min Yao[1,2,3 *]

[1] Faculty of Advanced Life Science, Hokkaido University, Sapporo 060-0810, Japan.
[2] Graduate School of Life Science, Hokkaido University, Sapporo 060-0810, Japan.
[3] Department of Pharmacology, Basic Medical College of Zhengzhou University, Zhengzhou, China

[*]Correspondence and requests for materials should be addressed to M.Y.
email:yao@castor.sci.hokudai.ac.jp

Materials and Methods

Cloning, expression, and purification of *Pa*LpxH

The *lpxH* gene was amplified from *P. aeruginosa* PAO1 genomic DNA using PCR with sense primer 5′-CCCCACATATGAGCGTCCTGTTCATCTCGG-3′ and antisense primer 5′-GGGGTCTCGAGGAGCGGGAAGGGCGCCTG-3′. The resulting PCR product was cloned into the *Nde*I and *Xho*I sites of pET-26b (Novagen), which was then used to transform *E. coli* B834(DE3)pLysS competent cells (Novagen).

Expression of *Pa*LpxH carrying a C-terminal hexahistidine tag was induced by the addition of 1 mM isopropyl-β-D-thiogalactopyranoside to cultures in Luria broth containing kanamycin (30 mg/L) after the OD_{600} reached 0.6 at 37 °C. Cell cultures were incubated with shaking for an additional 2 h and harvested by centrifugation. The following steps were performed at 4 °C unless otherwise noted. Cells from 3 L of induced culture were resuspended in suspension buffer containing 50 mM phosphate–citrate, pH 6.0; 20 mM MES, pH 6.0; 600 mM NaCl; 10% (w/v) sucrose; 5 mM 2-mercaptoethanol; 10 mM imidazole; and 0.1% (w/v) Triton X-100; and supplemented with 0.2 mg/mL lysozyme and 0.1 mg/mL DNase I. The suspended cells were lysed by sonication. Cell debris were removed by centrifugation at 45,000 ×*g* for 30 min at 10 °C. The supernatant was bound to a 6-mL bed volume of Ni Sepharose 6 Fast Flow (GE Healthcare) resin equilibrated in suspension buffer by gentle stirring for 30 min and then the Ni resin was collected by centrifugation at 2000 ×*g* for 2 min. After the supernatant containing unbound material was removed, 10 bed volumes of the suspension buffer were added to the resin for washing and gently stirred for 15 min. After the wash was repeated twice, the washed resin was packed into a chromatography column and attached to an AKTA FPLC system (GE Healthcare). The column was washed again with five column volumes of buffer *A*, which contained 20 mM phosphate–citrate, pH 6.0; 20 mM MES, pH 6.0; 300 mM NaCl; 5% (v/v) glycerol; 5 mM 2-mercaptoethanol; and 10 mM imidazole. *Pa*LpxH was eluted with a gradient of 10–400 mM imidazole. Eluted fractions were loaded onto a 5-mL HiTrap Heparin HP column (GE Healthcare) equilibrated with buffer *B*, which contained 20 mM phosphate–citrate, pH 6.0; 20 mM MES, pH 6.0; 300 mM NaCl; 5% (v/v) glycerol; and 1 mM dithiothreitol (DTT). The *Pa*LpxH bound to heparin was eluted with a gradient of 750–1350 mM NaCl. Fractions containing *Pa*LpxH were further purified by gel filtration on a HiLoad 26/60 Superdex 200 (GE Healthcare) column pre-equilibrated with buffer *C*, which contained 20 mM MES, pH 6.0; 200 mM NaCl; and 1 mM DTT supplemented with 5% (v/v) glycerol. The purified protein was concentrated to 14 mg/mL using a Vivaspin 20 10k ultrafiltration device (GE Healthcare) and stored at −80 °C. Site-directed mutagenesis of *Pa*LpxH (H10N) was performed using a Quick mutagenesis kit (Stratagene). The H10N mutant was expressed and purified using the same protocols as those used with wild-type *Pa*LpxH.

Crystallization

Initial crystallization trials were performed using the “Index” crystallization kit from Hampton Research, and initial crystals were obtained from several conditions. After optimization, all crystals were grown using the sitting-drop vapor-diffusion method at 20 °C. Each drop was prepared by mixing equal volumes of protein and reservoir solutions.

$P2_1$ crystals with lipid X and two Mn^{2+} were obtained using protein solution (buffer *C* containing 5.1 mg/mL *Pa*LpxH and 1.0 mM $MnCl_2$) and reservoir solution (0.2 M proline; 0.1 M HEPES, pH 7.6; and 8.5% PEG 3350). $P2_1$ crystals without Mn^{2+} were obtained using protein solution (buffer *C* containing 6.8 mg/mL *Pa*LpxH and 5% glycerol, 0.1 mM EDTA) and reservoir solution (0.2 M proline; 0.1 M HEPES, pH 7.4; 9.0% PEG 3350; and 5% glycerol), and the crystals were soaked in the reservoir solution containing 1 mM EDTA for 10 h. *C*2 crystals without Mn^{2+} were obtained using protein solution (buffer *C* containing 8.5 mg/mL *Pa*LpxH and 5% glycerol) and reservoir solution (131 mM DL-malic acid, pH7.0; 17.5% PEG 3350; and 10% glycerol). $P2_12_12_1$ crystals of H10N mutant with one Mn^{2+} were obtained using protein solution (buffer *C* containing 6.3 mg/mL *Pa*LpxH and 1.0 mM $MnCl_2$) and reservoir solution (0.2 M proline; 0.1 M MES, pH 6.7; and 12.5% PEG 3350). $P2_12_12_1$ crystals of H10N mutant without Mn^{2+} were obtained using protein solution (buffer *C* containing 8.5 mg/mL *Pa*LpxH H10N) and reservoir solution (0.2 M proline; 0.1 M HEPES, pH 7.0; and 10.0% PEG 3350).

Table S1. Data collection and refinement statistics (molecular replacement)

	$P2_1$_2Mn (peak)	$P2_1$_2Mn (remote)	$P2_1$_EDTA	$C2$_no-Mn	$P2_12_12_1$_H10N_1Mn	$P2_12_12_1$_H10N_no-Mn
PDB code		5B49	5B4A	5B4B	5B4C	5B4D
**Data collection statistics**†						
Beamline	SPring-8 BL41XU		PF BL5A	PF AR-NW12A	SPring-8 BL32XU	SPring-8 BL41XU
Wavelength (Å)	1.8925	1.0000	1.0000	1.0000	1.0000	1.0000
Space group	$P2_1$		$P2_1$	$C2$	$P2_12_12_1$	$P2_12_12_1$
Cell dimensions						
a, b, c (Å)	48.9, 108.8, 51.6	48.9, 108.9, 51.6	49.0, 108.8, 51.7	143.5, 42.5, 100.8	53.6, 98.0, 113.6	67.6, 87.9, 98.4
β (°)	92.7	92.7	92.3	125.1		
Resolution range (Å)	50 - 2.12 (2.00 - 2.12)	50 - 1.65 (1.71 - 1.65)	50 - 1.72 (1.78 - 1.72)	50 - 1.60 (1.66 - 1.60)	50 - 1.95 (2.02 - 1.95)	50 - 1.75 (1.81 - 1.75)
Unique reflections	30086 (2701)	64565 (6376)	57104 (5711)	65538 (6365)	43726 (4301)	59677 (5887)
Multiplicity	7.0 (5.7)	6.7 (6.6)	3.6 (3.6)	5.0 (4.8)	3.6 (3.6)	4.9 (4.8)
Completeness (%)	98.9 (89.8)	100 (100)	99.9 (100.0)	99.0 (97.0)	99.7 (99.8)	99.8 (100.0)
Mean I/sigma(I)	25.0 (9.75)	20.4 (2.23)	18.2 (2.01)	18.3 (1.97)	17.7 (2.23)	18.4 (2.13)
R_{sym} (%)	9.1 (17.2)	7.5 (48.5)	6.4 (54.2)	7.3 (57.5)	7.8 (45.1)	7.8 (55.1)
**Refinement statistics**‡						
R_{work}/R_{free}		0.152/0.177	0.160/0.188	0.161/0.190	0.171/0.214	0.178/0.202
Number of non-H atoms		4640	4636	4509	4200	4362
macromolecules		3942	3950	3891	3732	3752
ligands		118	108	102	26	18
water		580	578	516	442	592
Protein residues		486	487	481	462	465
RMS(bonds)		0.011	0.010	0.010	0.011	0.016
RMS(angles)		1.41	1.38	1.39	1.33	1.47
Ramachandran favored (%)		98	99	98	98	99
Ramachandran allowed (%)		2	1	2	2	1
Ramachandran outliers (%)		0	0	0	0	0
Average B-factor		24.1	24.6	20.5	32.9	27.9
macromolecules		22.8	23.5	18.9	31.9	26.5
ligands		26.2	24.0	25.2	44.6	47.2
solvent		32.6	32.1	31.3	40.6	36.3

†Values in parentheses are for the highest resolution shell. $R_{sym} = \{\Sigma_h \Sigma_i \mid I_{h,i} - \langle I_h \rangle \mid\} / \Sigma_h \Sigma_i I_{h,i}$, where $\langle I_h \rangle$ is the mean intensity of a set of equivalent reflections.

‡$R = \{\Sigma||F_{obs}| - |F_{calc}||\}/\Sigma|F_{obs}|$, where $|F_{obs}|$ and $|F_{calc}|$ are observed and calculated structure factor amplitudes, respectively. R_{free} was calculated for 5% randomly selected reflections of data sets that were not used in the refinement. R_{work} was calculated with remaining reflections.

Fig. S1

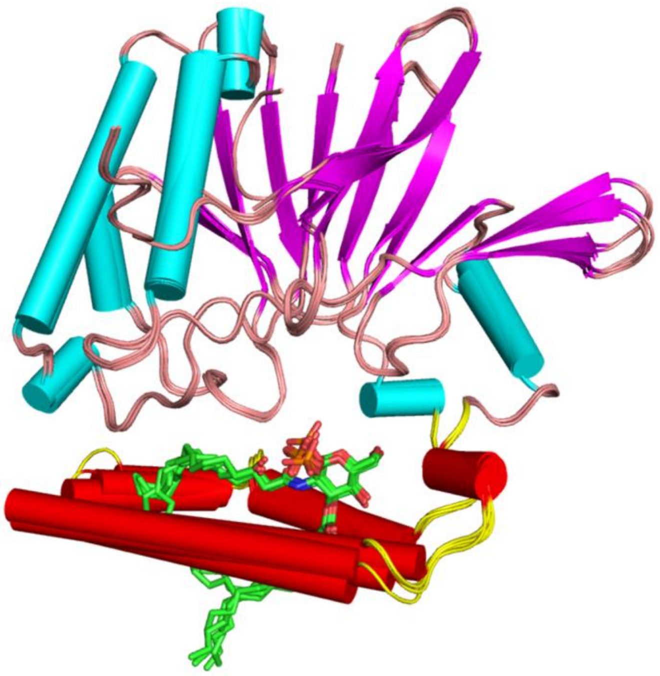

Fig. S1. Enzyme-product complex structures of *Pa*LpxH.

Superposition of six structures of *Pa*LpxH–lipid X complexes from three different crystals (*P*2_1 crystal with Mn^{2+}, *P*2_1 crystal with no Mn^{2+}, and *C*2 crystal with no Mn^{2+}, each containing two complexes in the asymmetric unit). Cartoon diagram is colored by secondary structure of each domain (catalytic domain: helix, cyan; sheet, magenta; loop, salmon pink; HI domain: helix, red; loop, yellow). While the six structures of *Pa*LpxH superpose well, the 3-acyl chains of lipid X show conformational variation.

Fig. S2

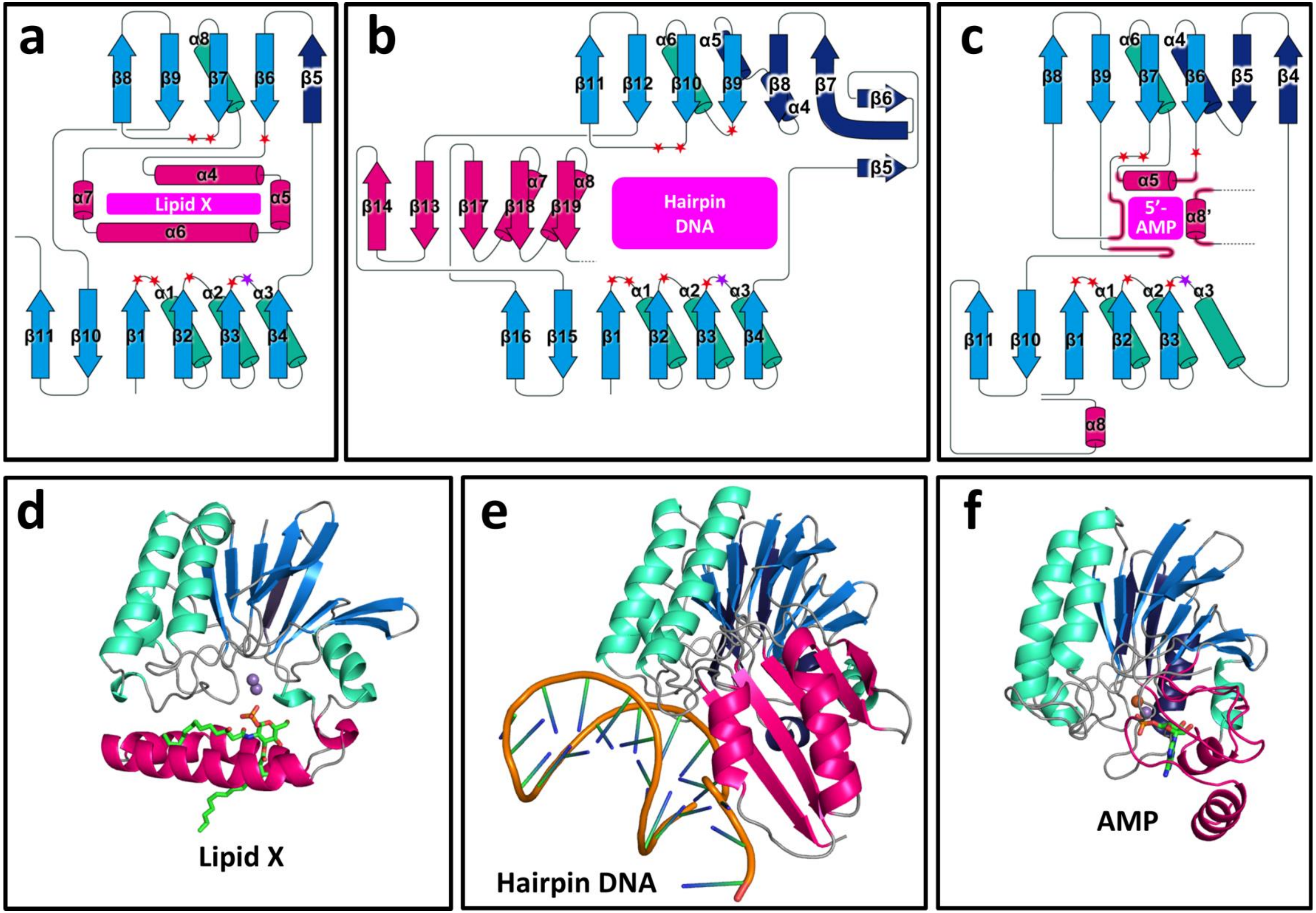

a
β8
β9
α8
β7
β6
β5
α4
α7
Lipid X
α5
α6
α1
α2
α3
β11
β10
β1
β2
β3
β4
b
α6
α5
β11
β12
β10
β9
β8
β7
β6
α4
β5
α7
α8
β14
β13
β17
β18
β19
Hairpin DNA
α1
α2
α3
β16
β15
β1
β2
β3
β4
c
α6
α4
β8
β9
β7
β6
β5
β4
α5
5′-AMP
α8′
α1
α2
α3
β11
β10
β1
β2
β3
α8
d
Lipid X
e
Hairpin DNA
f
AMP

Fig. S2. Comparisons of the secondary and tertiary structures of three MPEs.

a and d: LpxH (present study), b and e: Mre11 (multifunctional nuclease involved in DNA repair) **(1)**, c and f: Rv0805 (cyclic AMP phosphoesterase) **(2).** In the topology models of LpxH (a), Mre11 (b), and Rv0805 (c), the catalytic domains commonly present in all three proteins are depicted in light blue (β-strand) and green (α-helix). Other regions are in dark blue, whereas the substrate-binding domains are drawn in pink. Rv0805 does not have structurally independent substrate-binding domain but has a substrate-binding pocket between two subunits. The positions of the conserved residues coordinated to the two metal ions are marked with red stars. The residues important for enzymatic activity are marked with purple stars. In the tertiary structures of LpxH (d), Mre11 (e), and Rv0805 (f), the secondary structure elements are colored according to the topology models. The reaction products (lipid X and AMP) are shown as stick models and the substrate analog (hairpin DNA) is shown as a cartoon diagram.

Fig. S3

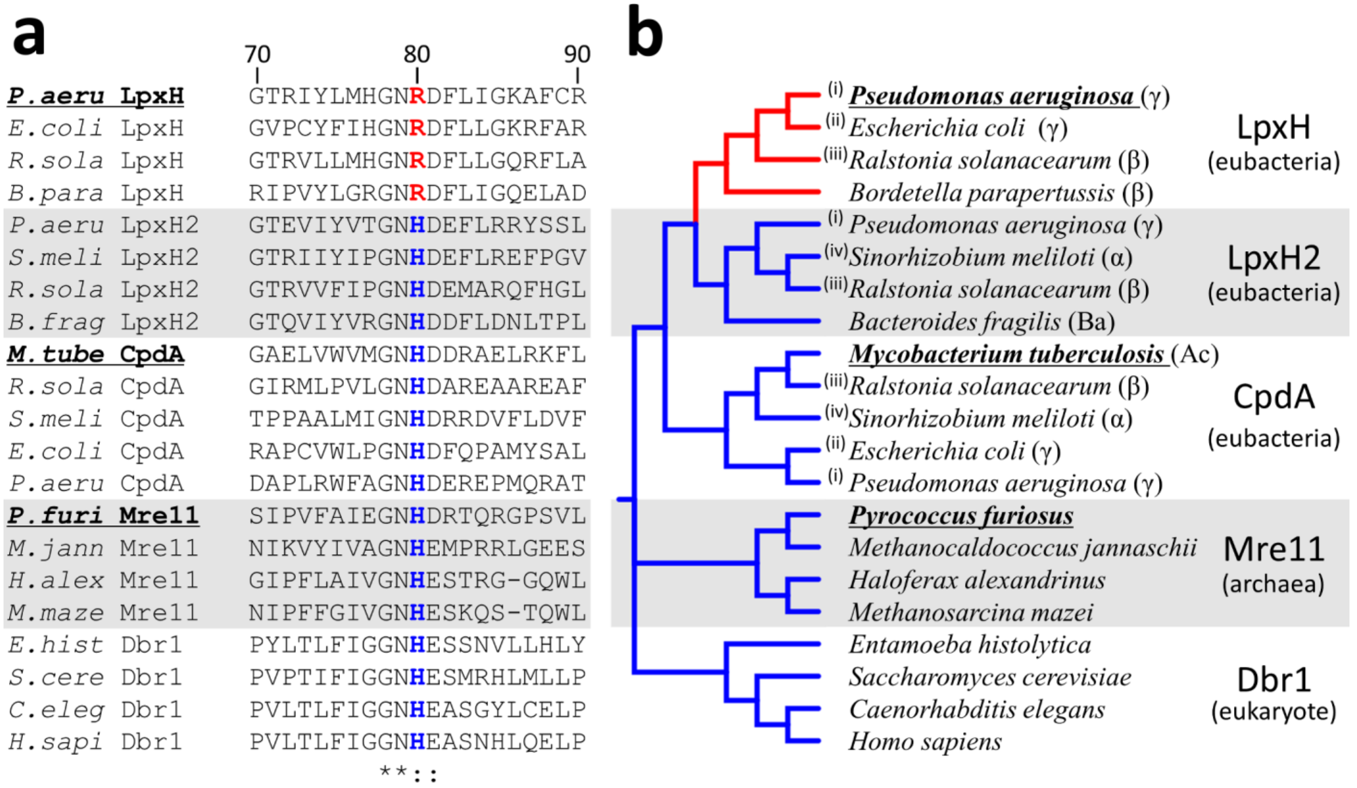

Fig. S3. Sequence alignment and phylogenetic tree of LpxH and other MPEs.

a. A multiple sequence alignment of MPEs from all domains of life was generated using Clustal Omega (3), and the region between Gly70 and Arg90 of *Pa*LpxH is shown. Note that the 80th position is His (blue, bold) for almost all MPEs with the single exception for LpxH, where it is replaced by Arg (red, bold). Proteins highlighted with bold letters and underlines are those shown in Fig. S2. b. Phylogenetic tree based on the multiple alignments in (A). In bacteria marked by i, ii, iii, and iv, more than one MPE is present. (α): alphaproteobacteria, (β): betaproteobacteria, (γ): gammaproteobacteria, (Ba): bacteroidetes, (Ac): actinobacteria.

Fig. S4

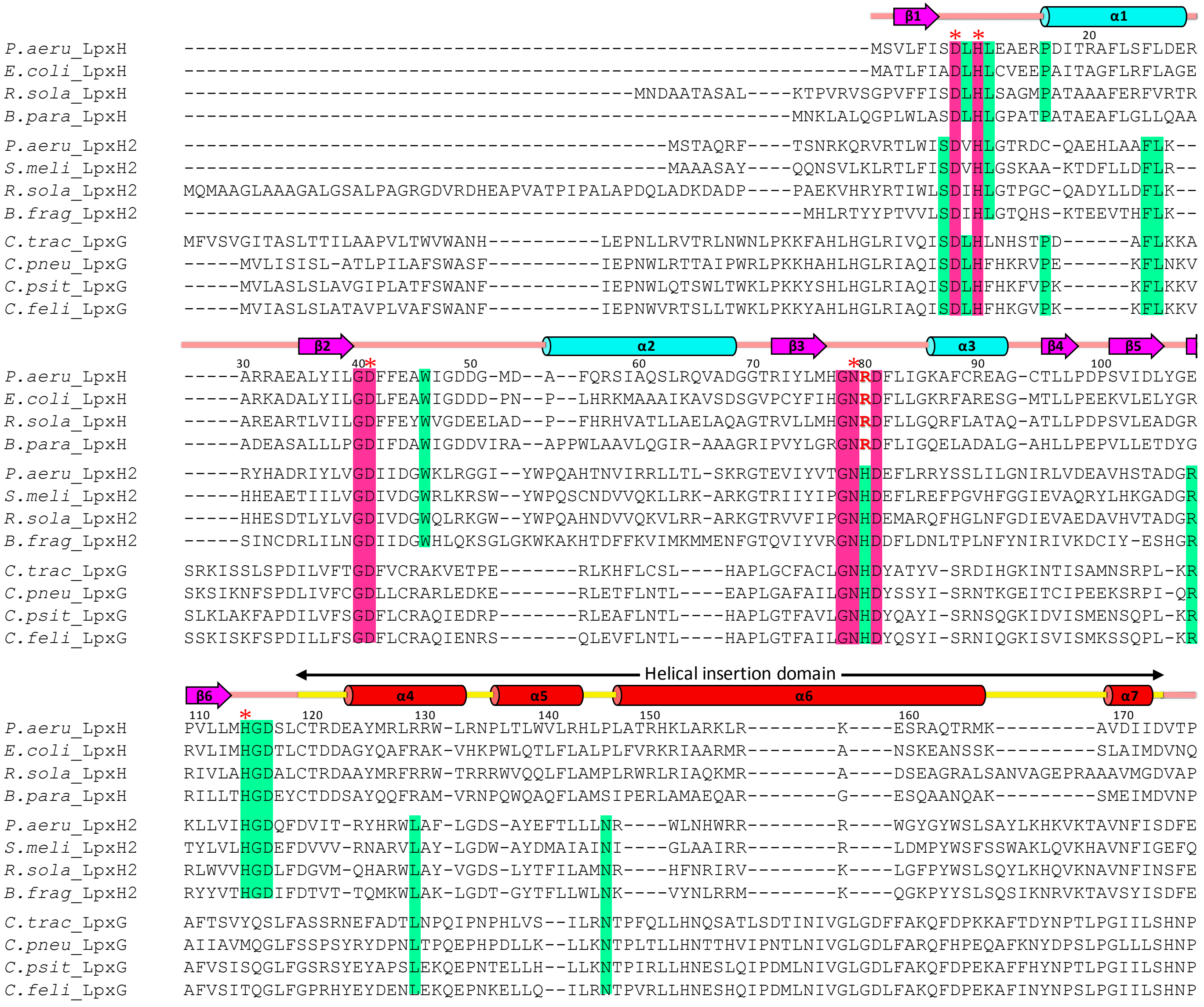

β1
α1
20
P.aeru_LpxH ---MSVLFISDLHLEAERPDITRAFLSFLDER
E.coli_LpxH ---MATLFIADLHLCVEEPAITAGFLRFLAGE
R.sola_LpxH --MNDAATASAL----KTPVRVSGPVFFISDLHLSAGMPATAAAFERFVRTR
B.para_LpxH --MNKLALQGPLWLASDLHLGPATPATAEAFLGLLQAA
P.aeru_LpxH2 ---MSTAQRF----TSNRKQRVRTLWISDVHLGTRDC-QAEHLAAFLK--
S.meli_LpxH2 ---MAAASAY----QQNSVLKLRTLFISDVHLGSKAA-KTDFLLDFLR--
R.sola_LpxH2 MQMAAGLAAAGALGSALPAGRGDVRDHEAPVATPIPALAPDQLADKDADP----PAEKVHRYRTIWLSDIHLGTPGC-QADYLLDFLK--
B.frag_LpxH2 ---MHLRTYYPTVVLSDIHLGTQHS-KTEEVTHFLK--
C.trac_LpxG MFVSVGITASLTTILAAPVLTWVWANH----------LEPNLLRVTRLNWNLPKKFAHLHGLRIVQISDLHLNHSTPD------AFLKKA
C.pneu_LpxG -----MVLISISL-ATLPILAFSWASF----------IEPNWLRTTAIPWRLPKKHAHLHGLRIAQISDLHFHKRVPE------KFLNKV
C.psit_LpxG -----MVLASLSLAVGIPLATFSWANF----------IEPNWLQTSWLTWKLPKKYSHLHGLRIAQISDLHFHKFVPK------KFLKKV
C.feli_LpxG -----MVIASLSLATAVPLVAFSWANF----------IEPNWVRTSLLTWKLPKKYAHLHGLRIAQISDLHFHKGVPK------KFLKKV
β2
α2
β3
α3
β4
β5
30
40
50
60
70
80
90
100
P.aeru_LpxH -----ARRAEALYILGDFFEAWIGDDG-MD--A--FQRSIAQSLRQVADGGTRIYLMHGNRDFLIGKAFCREAG-CTLLPDPSVIDLYGE
E.coli_LpxH -----ARKADALYILGDLFEAWIGDDD-PN--P--LHRKMAAAIKAVSDSGVPCYFIHGNRDFLLGKRFARESG-MTLLPEEKVLELYGR
R.sola_LpxH -----AREARTLVILGDFFEYWVGDEELAD--P--FHRHVATLLAELAQAGTRVLLMHGNRDFLLGQRFLATAQ-ATLLPDPSVLEADGR
B.para_LpxH -----ADEASALLLPGDIFDAWIGDDVIRA--APPWLAAVLQGIR-AAAGRIPVYLGRGNRDFLIGQELADALG-AHLLPEPVLLETDYG
P.aeru_LpxH2 -----RYHADRIYLVGDIIDGWKLRGGI--YWPQAHTNVIRRLLTL-SKRGTEVIYVTGNHDEFLRRYSSLILGNIRLVDEAVHSTADGR
S.meli_LpxH2 -----HHEAETIILVGDIVDGWRLKRSW--YWPQSCNDVVQKLLRK-ARKGTRIIYIPGNHDEFLREFPGVHFGGIEVAQRYLHKGADGR
R.sola_LpxH2 -----HHESDTLYLVGDIVDGWQLRKGW--YWPQAHNDVVQKVLRR-ARKGTRVVFIPGNHDEMARQFHGLNFGDIEVAEDAVHVTADGR
B.frag_LpxH2 -----SINCDRLILNGDIIDGWHLQKSGLGKWKAKHTDFFKVIMKMMENFGTQVIYVRGNHDDFLDNLTPLNFYNIRIVKDCIY-ESHGR
C.trac_LpxG SRKISSLSPDILVFTGDFVCRAKVETPE-------RLKHFLCSL----HAPLGCFACLGNHDYATYV-SRDIHGKINTISAMNSRPL-KR
C.pneu_LpxG SKSIKNFSPDLIVFCGDLLCRARLEDKE-------RLETFLNTL----EAPLGAFAILGNHDYSSYI-SRNTKGEITCIPEEKSRPI-QR
C.psit_LpxG SLKLAKFAPDILVFSGDFLCRAQIEDRP-------RLEAFLNTL----HAPLGTFAVLGNHDYQAYI-SRNSQGKIDVISMENSQPL-KR
C.feli_LpxG SSKISKFSPDILLFSGDFLCRAQIENRS-------QLEVFLNTL----HAPLGTFAILGNHDYQSYI-SRNIQGKISVISMKSSQPL-KR
Helical insertion domain
β6
α4
α5
α6
α7
110
120
130
140
150
160
170
P.aeru_LpxH PVLLMHGDSLCTRDEAYMRLRRW-LRNPLTLWVLRHLPLATRHKLARKLR--------K----ESRAQTRMK---------AVDIIDVTP
E.coli_LpxH RVLIMHGDTLCTDDAGYQAFRAK-VHKPWLQTLFLALPLFVRKRIAARMR--------A----NSKEANSSK---------SLAIMDVNQ
R.sola_LpxH RIVLAHGDALCTRDAAYMRFRRW-TRRRWVQQLFLAMPLRWRLRIAQKMR--------A----DSEAGRALSANVAGEPRAAAVMGDVAP
B.para_LpxH RILLTHGDEYCTDDSAYQQFRAM-VRNPQWQAQFLAMSIPERLAMAEQAR--------G----ESQAANQAK---------SMEIMDVNP
P.aeru_LpxH2 KLLVIHGDQFDVIT-RYHRWLAF-LGDS-AYEFTLLLNR----WLNHWRR--------R----WGYGYWSLSAYLKHKVKTAVNFISDFE
S.meli_LpxH2 TYLVLHGDEFDVVV-RNARVLAY-LGDW-AYDMAIAINI----GLAAIRR--------R----LDMPYWSFSSWAKLQVKHAVNFIGEFQ
R.sola_LpxH2 RLWVVHGDLFDGVM-QHARWLAY-VGDS-LYTFILAMNR----HFNRIRV--------K----LGFPYWSLSQYLKHQVKNAVNFINSFE
B.frag_LpxH2 RYYVTHGDIFDTVT-TQMKWLAK-LGDT-GYTFLLWLNK----VYNLRRM--------K----QGKPYYSLSQSIKNRVKTAVSYISDFE
C.trac_LpxG AFTSVYQSLFASSRNEFADTLNPQIPNPHLVS--ILRNTPFQLLHNQSATLSDTINIVGLGDFFAKQFDPKKAFTDYNPTLPGIILSHNP
C.pneu_LpxG AIIAVMQGLFSSPSYRYDPNLTPQEPHPDLLK--LLKNTPLTLLHNTTHVIPNTLNIVGLGDLFARQFHPEQAFKNYDPSLPGLLLSHNP
C.psit_LpxG AFVSISQGLFGSRSYEYAPSLEKQEPNTELLH--LLKNTPIRLLHNESLQIPDMLNIVGLGDLFAKQFDPEKAFFHYNPTLPGIILSHNP
C.feli_LpxG AFVSITQGLFGPRHYEYDENLEKQEPNKELLQ--ILRNTPVRLLHNESHQIPDMLNIVGLGDLFAKQFDPEKAFINYNPSLPGIILSHNP

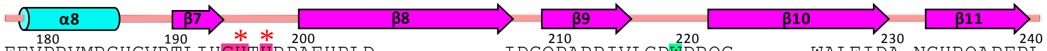


```
P.aeru_LpxH     EEVPRVMRGHGVRTLIHGHTHRPAEHPLD----------IDGQPARRIVLGDWDRQG------WALEIDA-NGHRQAPFPL---------
E.coli_LpxH     NAVVSAMEKHQVQWLIHGHTHRPAVHELI----------ANQQPAFRVVLGAWHTEG------SMVKVTA-DDVELIHFPF---------
R.sola_LpxH     EAVDALFKSAGIPLLIHGHTHRPRLHHEP--------------GGERWVLSDWDFDHAQP-RGSFLKLQD-GVLTVEPVTA---------
B.para_LpxH     ASVEAALREADVDVLVHGHTHRPARHVLS----------VDGRKRERWVLPDWDCDHADPPRGGWLVIDR-DGLQCFDLVEDED------

P.aeru_LpxH2    EAIAHECHKRGLDGVVCGHIHHAEIRRIE--------------GVDYLNCGDWVESC------TALIEHWDGSIQLYRLAEEQARLAAQA
S.meli_LpxH2    KVVTEEARRHDAQGVICGHIHHAVIEDID--------------GIRYINTGDWVESC------TAIAEHHDGTMELITWHQMRGDSAAGQ
R.sola_LpxH2    RVMTDEARRRGCDGVVCGHIHKAEIREID--------------GQLYCNDGDWVESL------SALVETMEGELQIVYWTHLLDAPPTSR
B.frag_LpxH2    KELVGLARAKKCDGVICGHIHHPANTFYE--------------DIHYLNSGDWVETL------SALTEDEDGNWTIRYFDSGLLKEDNHK

C.trac_LpxG     DTIHHL-QDYPGDVVFSGHSHGPQISLPWPKFANTITNKLSGLENPELARGLFSFPE--------------ES-RLLYVNRGLGGWKRIR
C.pneu_LpxG     DGITRL-QQYPGDFVLSGHSHGPQVTLSWPKFARKFFERLSGLENPYLARGYFVTKE---------------G-KQLYVNRGLGGLKRIR
C.psit_LpxG     DTVYQL-LDYPGDLIFSGHTHGPQISIPWPKFANRIMNKTSGLENPDLARGHFFFSE--------------GK-KQLYVNRGLGGFKRLR
C.feli_LpxG     DTVHQL-EDYPGDMVFSGHTHGPQISIPWPKFANKIMNKISGLENPELARGHFFFSG--------------GK-RQLYVNRGLGGLKRLR


P.aeru_LpxH     ------------------
E.coli_LpxH     ------------------
R.sola_LpxH     ------------------
B.para_LpxH     ------------------

P.aeru_LpxH2    AAVEPAA-----------
S.meli_LpxH2    RAGEAEAMPVRRLAPQAA
R.sola_LpxH2    RARRAAAAA---------
B.frag_LpxH2    EKQTIS-ITI---AS---

C.trac_LpxG     FCSPPEICLMRCLYEP--
C.pneu_LpxG     FCSPPEICYITCSYD---
C.psit_LpxG     FCSPPEICCIRCVYGS--
C.feli_LpxG     FCSPPEICCVRCVYES--
```

Fig. S4. Multiple sequence alignment of LpxH, LpxH2, and LpxG.

Conserved residues in all three orthologs are shaded in pink and those in two orthologs are in green. Residues that are likely to be involved in metal coordination are marked with red asterisks. The secondary structure of *Pa*LpxH is also shown at the top. Colors are as in Figure S1 (catalytic domain: helix, cyan; sheet, magenta; loop, salmon pink; HI domain: helix, red; loop, yellow). LpxH2 has an insertion sequence in the region corresponding to the HI domain of LpxH. However, the conserved residues in this insertion region are completely different between LpxH and LpxH2. LpxG seems to have an insertion sequence between completely conserved residues Asp125 and Gly256 (numbers are from *Chlamydia trachomatis* LpxG and correspond to Asp81 and Gly194 of *Pa*LpxH). However, the conserved residues between Asp81–Gly194 of LpxH and Asp125–Gly256 of LpxG are completely different. The species abbreviations are as follows: *P. aeru* (*Pseudomonas aeruginosa*), *E. coli* (*Escherichia coli*), *R. sola* (*Ralstonia solanacearum*), *B. para* (*Bordetella parapertussis*), *S. meli* (*Sinorhizobium meliloti*), *B. frag* (*Bacteroides fragilis*), *C. trac* (*Chlamydia trachomatis*), *C. pneu* (*Chlamydia pneumoniae*), *C. psit* (*Chlamydia psittaci*), and *C. feli* (*Chlamydophila felis*).

Fig. S5

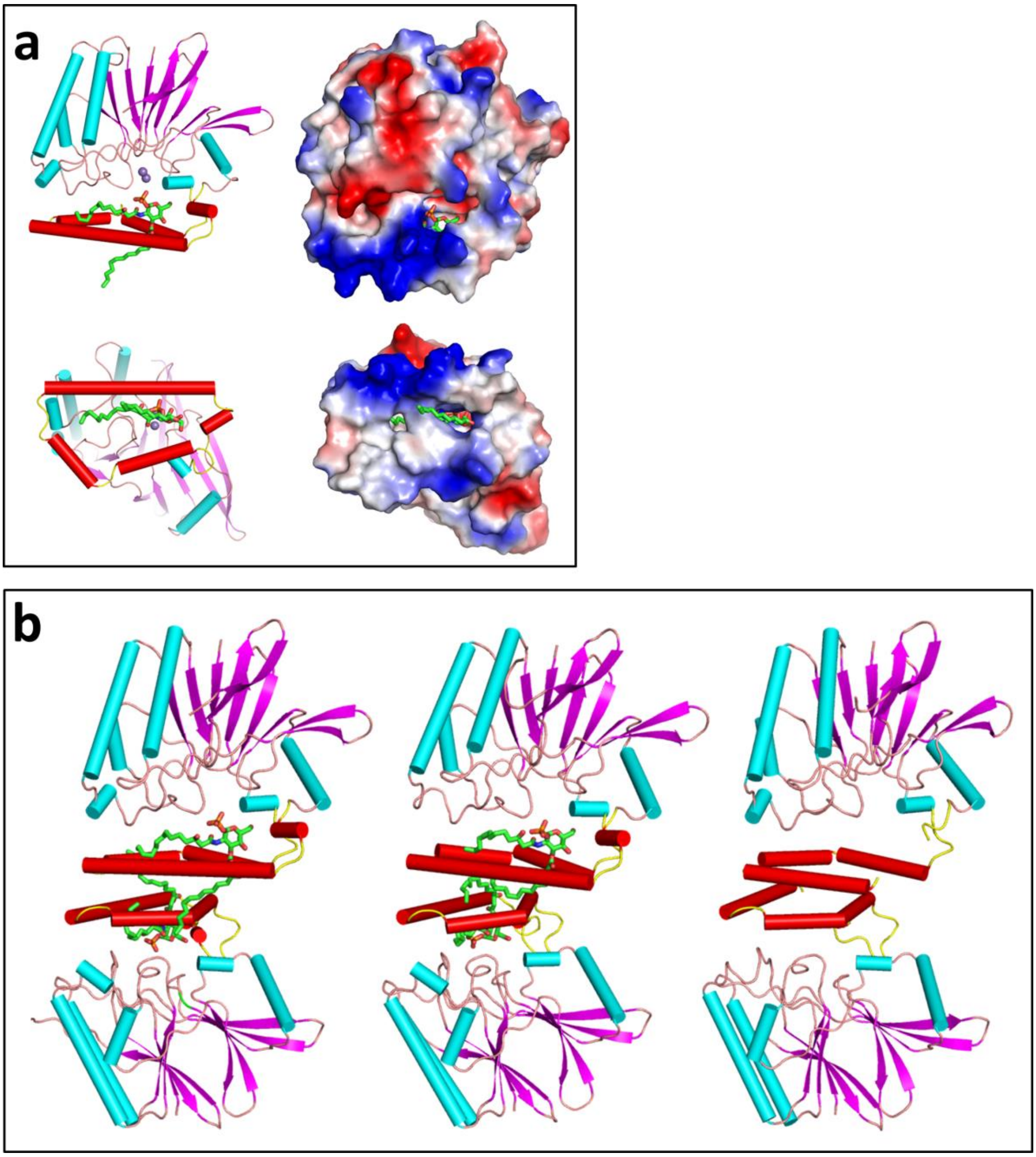

Fig. S5. Hydrophobic surface interaction of *Pa*LpxH

a. Orthogonal views of *Pa*LpxH with molecular surface representations.

Red and blue on the molecular surface represent negatively or positively charged potentials, respectively. The bottom surface of the HI domain is positively charged with a hydrophobic patch. b. Contacts between the two molecules in the asymmetric unit of *Pa*LpxH crystals, which mimic interactions with the membrane. From left to right, $P2_1$ crystal with 2 Mn^{2+}; $C2$ crystal with no Mn^{2+}; and $P2_12_12_1$ apo crystal with 1 Mn^{2+}.

Fig. S6

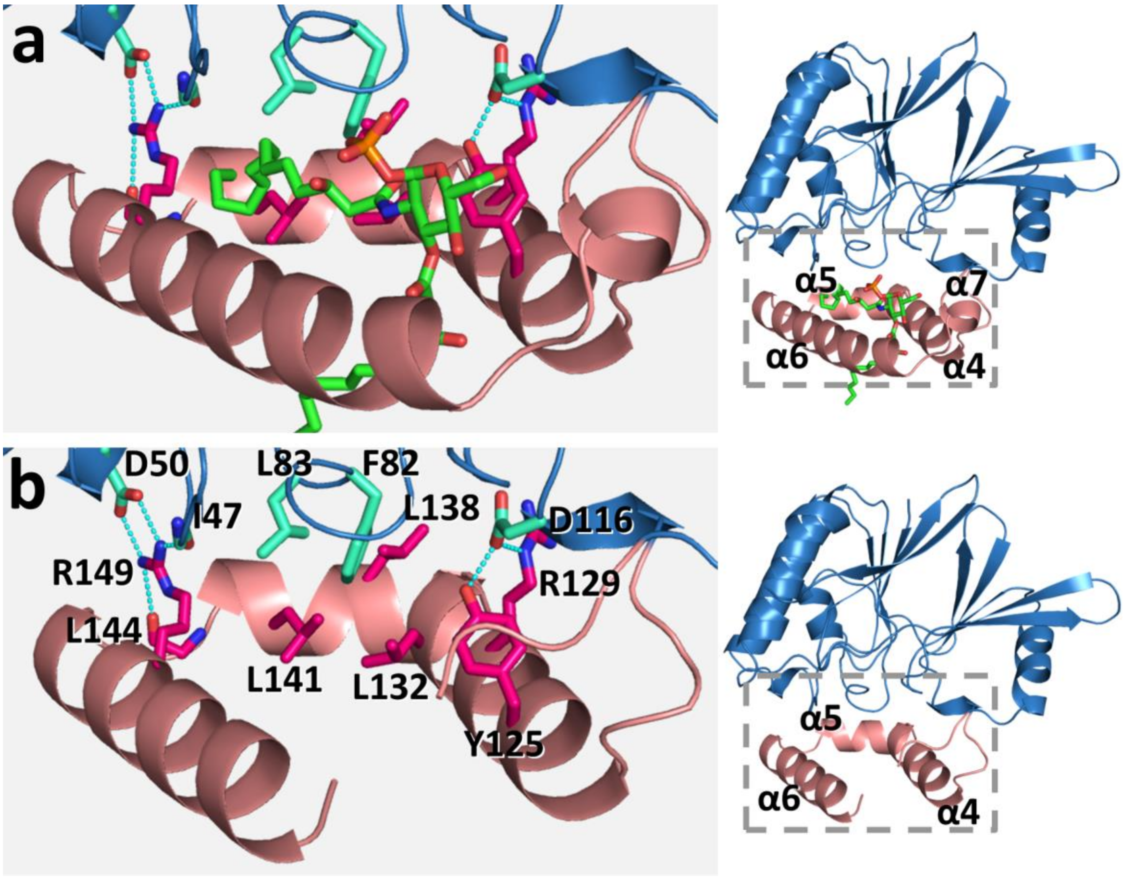

Fig. S6. Inter-domain interactions

Inter-domain interactions remain unchanged when the C-terminal half (α6–α7) of the HI domain undergoes a large conformational change upon lipid X binding or release.

a. Close-up view of the squared region of the EP complex. The catalytic domain is depicted in sky blue and the HI domain in salmon pink. Amino acid residues participating in inter-domain interactions are represented with stick models (residues in the catalytic domain, cyan; residues in the HI domain, magenta). Lipid X is also shown in stick. b. Close-up view of the squared region of the apo form. Colors are as in A.

References

1. Hopfner, K. P. *et al.* Structural biochemistry and interaction architecture of the DNA double-strand break repair Mre11 nuclease and Rad50-ATPase. *Cell* **105,** 473–85 (2001).
2. Podobnik, M. *et al.* A mycobacterial cyclic AMP phosphodiesterase that moonlights as a modifier of cell wall permeability. *J. Biol. Chem.* **284,** 32846–57 (2009).
3. Sievers, F. *et al.* Fast, scalable generation of high-quality protein multiple sequence alignments using Clustal Omega. *Mol. Syst. Biol.* **7,** 539 (2011).
